# Electrospray Mediated Localized and Targeted Chemotherapy in a Mouse Model of Lung Cancer

**DOI:** 10.3389/fphar.2021.643492

**Published:** 2021-04-20

**Authors:** Paulius Ruzgys, Stephan Böhringer, Ayse Sila Dokumaci, Yvonne Hari, Christian M. Schürch, Frido Brühl, Stefan Schürch, Sönke Szidat, Carsten Riether, Saulius Šatkauskas, Thomas Geiser, David Hradetzky, Amiq Gazdhar

**Affiliations:** ^1^ Department of Pulmonary Medicine, University Hospital Bern, Bern, Switzerland; ^2^ Department of Biomedical Research, University of Bern, Bern, Switzerland; ^3^ Biophysical Research Group, Faculty of Natural Sciences, Vytautas Magnus University, Kaunas, Lithuania; ^4^ Institute for Medical Engineering and Medical Informatics, School of Life Sciences, University of Applied Sciences and Arts Northwestern Switzerland, Muttenz, Switzerland; ^5^ Magnetic Resonance Spectroscopy and Methodology, Department of Clinical Research, University of Bern, Bern, Switzerland; ^6^ Department of Chemistry and Biochemistry, University of Bern, Bern, Switzerland; ^7^ Institute of Pathology, University of Bern, Bern, Switzerland; ^8^ Department of Medical Oncology, Inselspital Bern University Hospital, University of Bern, Bern, Switzerland

**Keywords:** localized and targeted therapy, electrospray, novel cancer treatment, coulomb repulsion, localized tumor treatment

## Abstract

**Background:** An advanced stage, centrally localized invasive tumor is a major cause of sudden death in lung cancer patients. Currently, chemotherapy, radiotherapy, laser ablation, or surgical resection if possible are the available state-of-the-art treatments but none of these guarantee remedy or long-term relief and are often associated with fatal complications. Allowing localized chemotherapy, by direct and confined drug delivery only at the tumor site, could be a promising option for preoperative down staging or palliative therapy. Here we report the localized and targeted application of intra tumor delivery of chemotherapeutics using a novel device based on the principle of electrospray.

**Methods:** C57BL/6J mice were injected with Lewis lung carcinoma cells subcutaneously. After 15 days, the animals were anesthetized and the tumors were exposed by skin incision. Tumors were electrosprayed with 100 µg cisplatin on days 0 and 2, and tumor volumes were measured daily. Animals were sacrificed on day 7 after the first electrospray and tumors were analyzed by immunohistochemistry.

**Results:** In this proof-of-concept study, we report that the tumor volume was reduced by 81.2% (22.46 ± 12.14 mm^3^) after two electrospray mediated Cisplatin deliveries, while the control tumor growth, at the same time point, increased by 200% (514.30 ± 104.50 mm^3^). Moreover, tunnel and Caspase-3 positive cells were increased after Cisplatin electrospray compared to other experimental groups of animals.

**Conclusion:** Targeted drug delivery by electrospray is efficient in the subcutaneous mouse model of lung cancer and offers a promising opportunity for further development toward its clinical application.

## Introduction

It is estimated that by 2030 lung cancer will be the 6th most common cause of death, affecting 1.6 million people worldwide. It is the most common cancer that leads to death in men (17%) and the third most common in women (13%) (Siegel et al., 2020). Smoking has been identified as the major cause of lung cancer with the highest incidence rate reported in North America, Europe, and East Asia. Moreover, with increasing populations now living in polluted metropolitan regions globally, the incidence of lung cancer is also increasing, particularly in non-smokers (Cheng et al., 2016; De Groot et al., 2018). Despite advances in diagnosis and therapy, the 5-year survival rate of patients with lung cancer is only 18% (Lu et al., 2019). Due to invasive tumor growth, 30% of all lung cancer patients suffer from a central airway obstruction (CAO), which is one of the major problems physicians face when caring for these patients. Patients develop respiratory distress and post stenotic pneumonia leading to suffocation and death (Ernst et al., 2004). Most patients who develop CAO are inoperable with no real or long lasting safe therapeutic options (Ernst et al., 2004; Lee et al., 2015). There is therefore a compelling need for successful lung cancer treatment. Recent advances, in the treatment of lung cancer are promising and several targeted therapies have been tested and reported on with mixed results. Targeted approaches based on immunotherapy are very expensive and have low efficiency (Thunnissen et al., 2014; Reck et al., 2016). This low efficiency is because current approaches rely on surface marker recognitions that are situated on the tumor cells, yet the therapeutics are administered systemically (Petrosyan et al., 2012; Chan and Hughes, 2015). Furthermore, heterogeneity of the tumor surface markers and the complexity of tumor stroma makes targeting approaches even more difficult. Additionally, selective delivery of chemotherapeutic drugs, only to tumor cells, is a major challenge in cancer chemotherapy. Various attempts have therefore been made that mainly rely on nanoparticle-based technology (Xin et al., 2017). The nanomedicine-based targeting approaches depend either on passive drug targeting using liposomes and polymers, or active drug targeting by ligands that are attached to drugs, acting as homing devices for binding to receptors expressed on the surface of the tumor cells. Many studies were performed, testing both methods in various kinds of tumors in different organs with mild effects (Lammers et al., 2008; Lammers et al., 2012). It is worth mentioning that the systemic route of administration was applied in all these studies. Interestingly, injecting chemotherapeutical drugs, locally, into the invasive lung tumor have been attempted (Celikoglu et al., 2010; Hohenforst-Schmidt et al., 2013); however, no randomized clinical studies have been reported to elucidate its safety and efficacy. Therefore, a promising approach could be to deliver therapeutic agents locally, at the site of the tumor, with drug delivery methods that utilize a physical force to achieve direct intracellular delivery gaining maximal therapeutic effect and avoiding systemic side effects. There have been attempts made for the local delivery of genes and chemotherapeutics in the past using physical methods like sonoporation, a method based on ultrasound in a mouse tumor model (Yuh et al., 2005; Iwanaga et al., 2007); however, due to technical limitations, this method could never be applied for clinical application. Another method of localized chemotherapy using electric pulses termed as electrochemotherapy is currently used for treating primary skin tumors (Caracò et al., 2013), metastatic skin tumors (Macri et al., 2014; Cabula et al., 2015), and head and neck tumors (Mevio et al., 2012; Bertino et al., 2016). Although electrochemotherapy has shown promising results when applied on external tumors, it has never been used for internal visceral tumors and invasive lung tumors. Therefore, a concept of local intracellular drug delivery that can be easily applied within the bronchus lumen, to target the invasive tumor to arrest tumor growth, could be a promising approach. We introduce the novel concept―the electrospray mediated targeted drug delivery method―using Cisplatin, to potentially treat invasive tumors in the bronchus. Electrospray, also known as electrohydrodynamic jetting, is based on the principle of Coulomb repulsion; under the influence of high voltage the flowing liquid breaks into small droplets that are then accelerated toward a counter electrode, which is in contact with the target tissue during delivery (Hradetzky et al., 2012; Boehringer et al., 2018). The accelerated droplets easily penetrate inside the cells facilitating drug delivery. The technical design and the proof-of-concept both *in vitro* and *in vivo* were demonstrated using electrospray mediated eGFP gene transfer (Boehringer et al., 2018). In the current study, we investigate the effect of electrospray mediated localized chemotherapy using Cisplatin on the mice subcutaneous tumor model of lung adenocarcinoma *in vivo*.

## Materials and Methods

### *In vitro* Electrospray

Cell lines and drugs: For *in vitro* and *in vivo* studies, Lewis lung carcinoma cells (LLC cells (ATCC; United States) were obtained as generous gift from Prof. Adrian Ochsenbein (Department of Biomedical Research University of Bern Switzerland). LLC cells were grown in DMEM medium containing 10% fetal bovine serum (Life technologies, United States). Cisplatin (Sigma Aldrich, United States) was used at a concentration of 1 nM for *in vitro* experiments and at a 3.3 mM (1 mg/ml) concentration for *in vivo* application. For *in vitro* experiments Cisplatin was diluted in 370 mM sucrose. For the *in vivo* study Cisplatin was used at a concentration of 0.2 mg in the total volume of 100 µl.

Tandem Mass Spectrometry of Cisplatin: The DNA hexamer 5′-TTCGGC-3′ (Microsynth, Balgach, Switzerland) was dissolved in water to obtain a 1 mM stock solution. Cisplatin samples were collected before and after the electrospray process and incubated over night at 37°C with equimolar amounts of the DNA hexamer. The incubated samples were diluted to 25 µM in a solvent mixture containing water, acetonitrile, and triethylamine at a ratio of 49:49:2. The mass spectrometric analysis was performed on a LTQ Orbitrap XL instrument (Thermo Fisher Scientific, Bremen, Germany) equipped with a nano-electrospray ionization source. Analyses were performed in the negative ion mode. The triply charged cisplatin adduct with m/z 669 was selected as the precursor ion and subjected to collision-induced dissociation (CID) using nitrogen as the collision gas.

*In vitro* electrospray on Lewis lung carcinoma cells: Lewis lung carcinoma (LLC) cells were grown in DMEM media (Life Technologies, United States) in 10% FCS (Life Technologies, United States) and 1% Penicillin/Streptomycin (Life Technologies, United States). For *in vitro* experiments, 24 well plates (BD Biosciences, United States) were coated with purified bovine collagen solution (PureCol Biomatrix, United States) at a concentration of 60 μg/ml 1 × 10^5^ cells were plated in the center of the collagen coated wells, suspended in culture media in a volume of 10 µL. To facilitate attachment, cells were incubated for 30 min at 37°C and 5% CO_2_ and then replenished with 500 µL growth medium. Cells grew as a monolayer in the center of the plate and were placed under the electrodes during electrospray procedure. Before electrospray was performed, cells were checked for homogenous distribution in the center of the well under the microscope.

*In vitro* electrospray: The electrospray procedure was performed using the prototype device previously developed and described (Boehringer et al., 2018). For all *in vitro* purposes, Cisplatin was used at a concentration of 1 nM dissolved in 370 mM sucrose. The electrospray parameters were as follows; 3 kV voltage, working distance (distance between tip of capillary and the target cells) 4 mm, flowrate 20 μL/min, and s volume of 25 µL of chemotherapeutical drug per suspension. All parameters are based on previous a study (Boehringer et al., 2018).

*In vitro* electrospray was performed four times, with 25 µL per treatment, and with a 30 s lag between each electrospray. After electrospray, cells were incubated for 10 min, media was replenished, and cells were placed in the incubator. After 24 h of incubation, Annexin V/PI staining and flow cytometry measurements were performed as described below. Three independent experiments were performed in triplicate.

Cisplatin uptake *In vitro*: Three different concentrations of Cisplatin were used (0.01 mg/ml, 0.1 mg/ml and 1 mg/ml), either *in-vitro* electrospray or conventional (i.e., without electrospray), and Cisplatin treatment was performed on LLC cells with 75 µL Cisplatin. Cells were divided in two sets and different washing procedures were performed. In one set, the cells were washed twice with Phosphate-buffered saline (PBS, 500 µL each) and twice with water (500 µL each), whereas washing was omitted for the second set. As controls, untreated LLC cells were analyzed, and the concentration of the used Cisplatin stock solution was measured for quantification of the recoveries. Ten minutes after electrospray, samples were digested in 0.5 ml 65% nitric acid (HNO_3_) at 95 C for 2 h and then diluted 1:20 with water. Samples without the washing step and the Cisplatin stock solution were further diluted at 1:500 with 3% HNO_3_. Gold (Au) was added as the internal standard and Platinum was analyzed by ICP-MS (inductively coupled plasma mass spectrometry) with a Varian 820-MS (Varian, Santa Clara, CA,United States), without the application of a collision gas to the sampler cone. Three independent experiments were performed in triplicate.

Annexin PI staining and analysis by FACS: Annexin V/PI staining was done using the FITC annexin V apoptosis detection kit with PI (Biolegend, United States), following the manufactures protocol. Flow cytometry measurements were done with the LSR II SORP H274 system (BD Biosciences, United States). A 488 nm blue laser was used for excitation. For emission measurements two filters were used, wavelength 585/15 was used for PI and Alexa Fluor 488 filter 525/50 was used for Annexin V. Data was analyzed with the single cell analysis software FLOWJO (FlowJo LLC, BD Bioscience, Canada). The positive control for the necrotic cell was made by freezing cell suspension in -80 °C for 20 min. The positive control for the apoptotic cell was done by incubating cells in 4% PFA for 30 min. Three independent experiments were performed in triplicate.

### *In vivo* Electrospray

Animals: C57BL/6J adult male mice were obtained from Janvier labs (France). The animals were kept *ad libitum*. Experiments were performed in accordance with the standards of the European Convention of Animal Care. The study protocol was approved by the Cantonal and University of Bern Animal Study Committee (BE 99/14).

Subcutaneous Tumor model: Lewis lung carcinoma (LLC) cells were grown in DMEM media (Life technologies, United States) in 10% FCS (Life technologies, United States). The animals were injected with 2 × 10^6^ LLC cells suspended in 100 µL of 1xPBS, subcutaneously, on both flanks. Tumor volume was measured using a caliper and experiments were performed after 15 days, as explained below.

Analgesia and Anesthesia: Mice were injected with buprenorphine (0.1 mg/kg) subcutaneously (sc) 30 min before surgery. For anesthesia, Midazolam (Dormicum) 5 mg/kg, Medetomidine (Domitor) 0.5 mg/kg, and Fentanyl (Fentanyl-Janssen) 0.05 mg/kg were administered intraperitoneally (0.05 ml per 20 gm mice). To protect the eyes of the mice, Bepanthen (5% Dexpanthenol) was applied before the procedure.

Electrospray mediated chemotherapy: The animals were divided into five groups with n = 5 in each group; (a) Control group (no drug), (b) Cisplatin local injection (no electrospray), (c) NaCl local injection (no electrospray), (d) Electrospray only, and (e) Cisplatin + electrospray. Analgesia and anesthesia were administered as described and the surgical procedure was performed. The tumor was exposed by a skin incision and the electrode of the electrospray device was placed on the tumor surface for electrospray. For *in vivo* electrospray, the following parameters were applied: voltage 3 kV, with working distance of 4 mm, and flowrate of 100 μL/min. Each tumor was electrosprayed four times at four different places, with 25 µL of the drug for each spray, and with a 30 s lag time between the spray. After electrospray, the skin was sutured using VICRYL 4.0 (Ethicon, Inc. United States). Mice were injected with antagonist subcutaneously (sc) for reversal of the anesthesia as described above. Analgesic buprenorphine (0.1 mg/kg) (sc) was administered daily. A second electrospray was performed two days later. Tumor volumes were measured every day using a caliper. Seven days after the first electrospray, the animals were euthanized by CO_2_ inhalation and tumors were excised and collected for analysis.

### Assessment

Caliper measurements of subcutaneous tumors: Mice were anesthetized by inhaling isoflurane in a glass box. Mice tumor volumes were evaluated by measuring the height, width, and length of the tumor using a caliper. The tumor shape was considered to be a rotary symmetric ellipsoid, and the volume was calculated using the formula Volume= (3.14*Height*Width*Length)/6 (Kersemans et al., 2013). The calculated volume of the first day was normalized to 100%, representing an initial relative tumor volume, and all subsequent volumes were divided by the initial tumor volume representing the fraction of tumor volume in %, to demonstrate the dynamic change in tumor volume.

Immunohistochemistry (IHC): Tumors were fixed in 4% neutral buffered formalin, embedded in paraffin. IHC staining was performed on a Leica BOND RX automated immunostainer (Leica Biosystems). Thin sections (1–2 µm) of tumors were pre-treated by boiling at 100°C in citrate buffer, pH 6.0. Sections were then stained at room temperature in Bond primary antibody diluent (Leica Biosystems) for 30 min using the rabbit anti-mouse cleaved caspase-3 (Asp175) (clone 5A1E, Cell Signaling Technologies, catalog no. 9664), dilution 1:100. Visualization was performed using the Bond Polymer Refine DAB Detection kit (Leica Biosystems, catalog no. DS9800) according to the manufacturer’s instructions. Images were captured using the 3D HISTECH slide scanner. Analysis of IHC was performed by two blinded surgical pathologists (C.M.S. and F.B.).

Tunnel assay: Tunnel assay was performed on paraffin embedded tissue using the Tunnel assay kit HRP-DAB (Abcam, United States), following the protocol provided. Total stained cells per area at high power field in three separate fields were counted using image J (Image J 1.51n, NIH, United States).

Statistics: *In vitro* experiments were done in triplicate and all the experiments were performed three times. Data is presented as mean ± SEM, and the student t test for *in vitro* data, or multiple comparison using ANOVA and Bonferroni correction, were performed using Graph Pad prism 7.0 (Graphpad software, United states).

## Results

### Electrospray Application of Cisplatin Does Not Change the Drug’s Mechanism of Action

**FIGURE 1:**
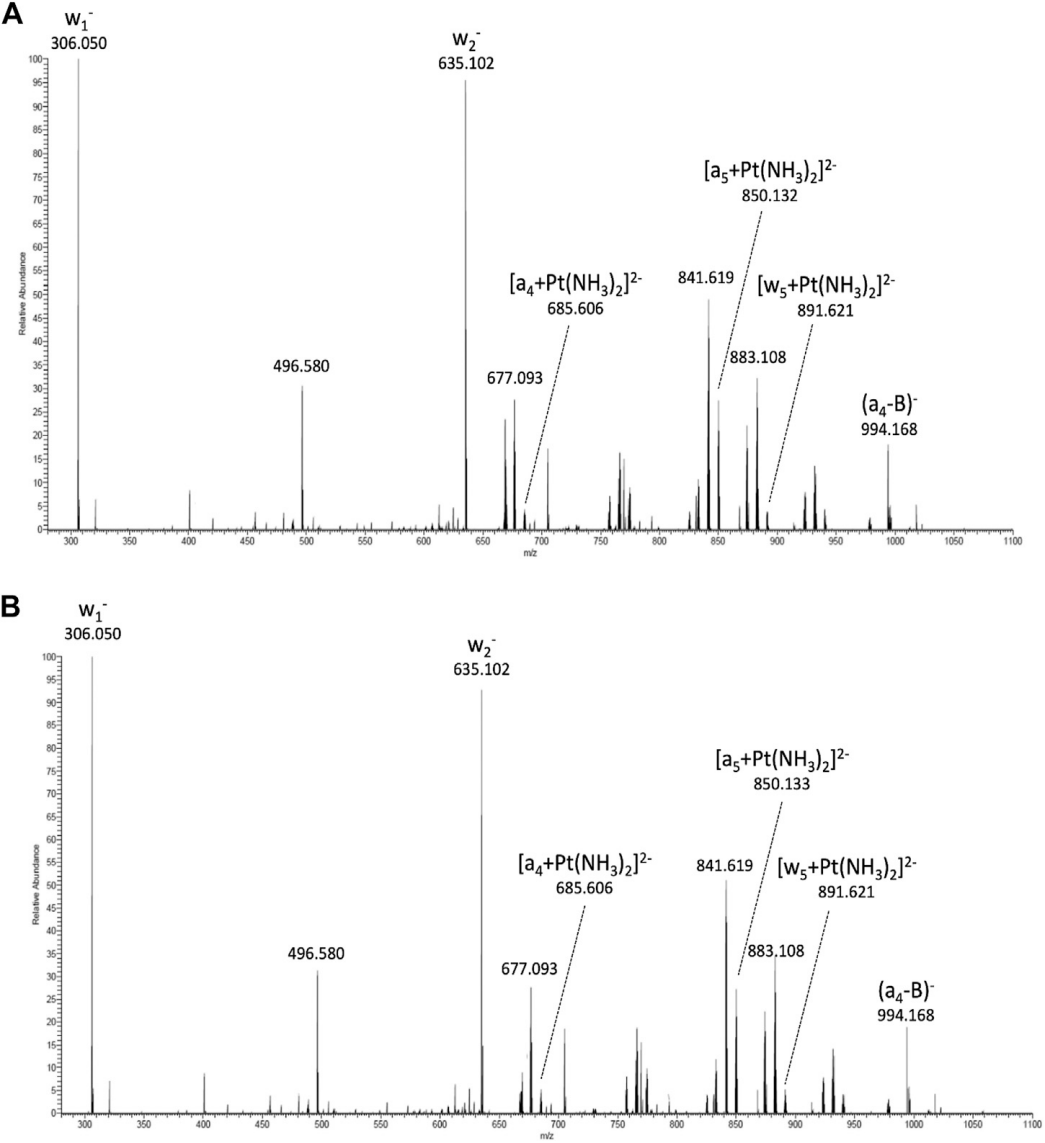
MS/MS spectra obtained by collision-induced dissociation of the triply charged platinated hexadeoxynucleotide precursor ions (m/z 669), which basically show the identical fragment ions and give evidence for adduct formation (= unchanged drug activity). Adduct formation and DNA binding sites by naïve Cisplatin **(A)** Cisplatin after electrospray **(B)**.

A potential cellular target of cisplatin is DNA. To study the effect of the electrospray process on the activity of the drug, cisplatin samples collected before and after electrospray were incubated with the DNA hexamer 5′-TTCGGC-3′ and subsequently analyzed by tandem mass spectrometry. Activation of selected precursor ions in the gas-phase, results in cleavage of the phosphate backbone, thus, generating sequence-specific fragment ions (Wu, 2004) (Schürch, 2016). The triply charged DNA-cisplatin adducts ((TTCGGC + cisPt)^3−^, m/z 668.6) were selected as the precursor ions. Mass spectrometric data indicates identical adduct formation in both samples treated with cisplatin solution before (Figure 1A) and after electrospray (Figure 1B). Both spectra show the peaks of fragment ions that indicate the binding of cisplatin to the vicinal guanine nucleobases (Nyakas et al., 2009). Binding of cisplatin promotes the cleavage of the 3′-C-O bond adjacent to the GG base pair and the subsequent release of cytidine monophosphate (w_1_^−^ fragment ion at m/z 306.05) from the 3′-end. The complementary fragment is observed as the doubly charged (TTCGG + cisPt)^2−^ ion at m/z 850.13. Besides these main fragment ions, indicative of adduct formation of cisplatin with the oligonucleotide, the binding of the drug is reflected by further platinum-containing ions generated by the loss of nucleobases from either end of the oligonucleotide (e.g. (TCGGC + cisPt)^2−^ at m/z 891.63 and (TTCG + cisPt)^2−^ at m/z 685.61).

The preferred binding site within the DNA hexamer was found to be the vicinal guanine nucleobases, which is consistent with previous studies. Consequently, the electrospray process neither affects the ability of cisplatin to form adducts, nor changes the preferred binding site on a DNA oligonucleotide.

#### Electrospray Increases Intracellular Uptake of Cisplatin *in vitro*

**FIGURE 2:**
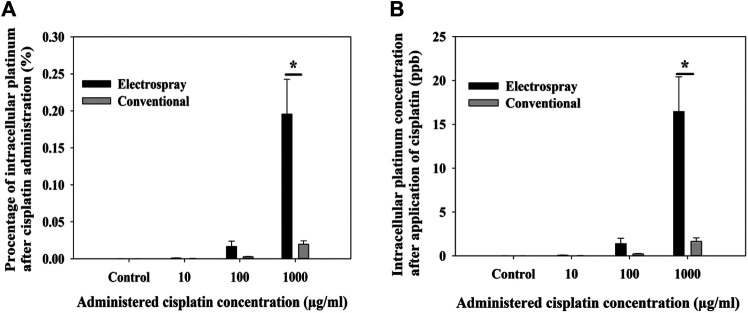
Intracellular uptake of Cisplatin; percentage of delivered Cisplatin that entered the cells **(A)** and amount of intracellular Cisplatin **(B)** after electrospray mediated Cisplatin delivery *in vitro*. Data presented as mean ± SEM, *p* < 0.05*, *p* < 0.001 **, *p* < 0.0001 ***.

Electrospray increases intracellular uptake of Cisplatin *in vitro*: Cisplatin 1 mg/ml was used for experiments. To quantify the amount of intracellular Cisplatin uptake after electrospray the LLC cells were treated with 75 μg, 7.5 µg of 0.75 µg (75 µl of volume) of Cisplatin delivered by electrospray or the cells were incubated with the same concentration of Cisplatin (conventional cell culture). The percentage of intracellular platinum was (412.2*10^−6^% ± 48.317*10^−6^%) when cells were incubated with Cisplatin at a concentration of 1,000 μg/ml, however when electrospray mediated Cisplatin, a delivery was performed, and the concentration increased to (1.033*10^−3^% ± 113*10^−6^%) (*p* ˂ 0.001) (Figure 2A). Moreover, intracellular concentration of platinum was significantly increased after electrospray mediated delivery (16.5 ± 3.9%) (*p* ˂ 0.001) (Figure 2B).

### Decreased Cell Viability After *in vitro* Electrospray

**FIGURE 3:**
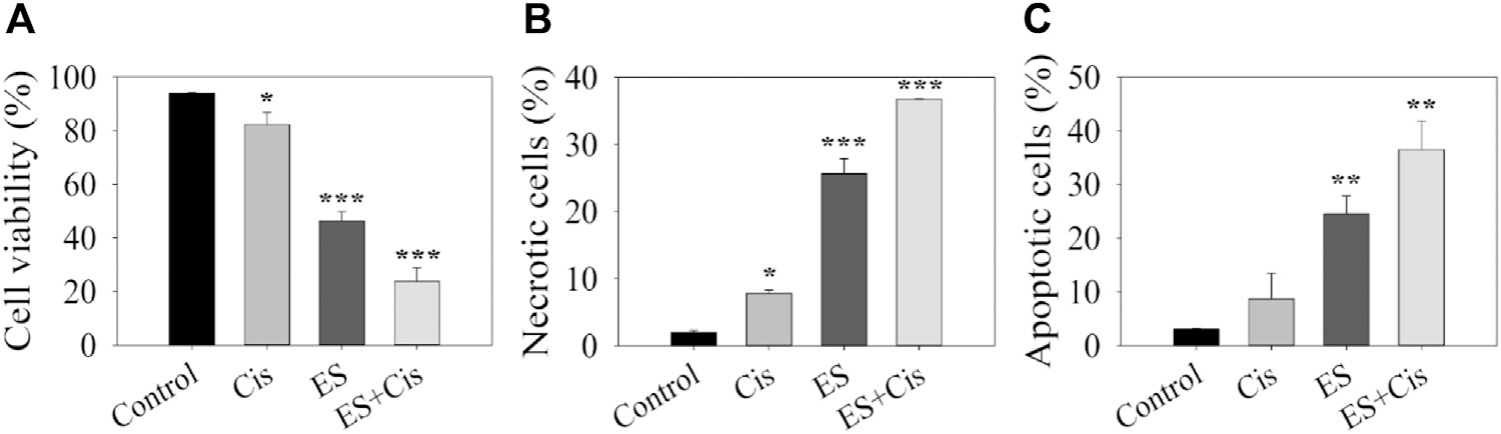
Effect of Cisplatin alone and electrospray Cisplatin delivery on cell viability **(A)**, necrosis **(B)** and apoptosis **(C)**. Graph showing Flow cytometry gating strategy for Annexin V/PI. Data presented as mean ± SEM, *p* < 0.05*, *p* < 0.001**, *p* < 0.0001***.

**FIGURE 4:**
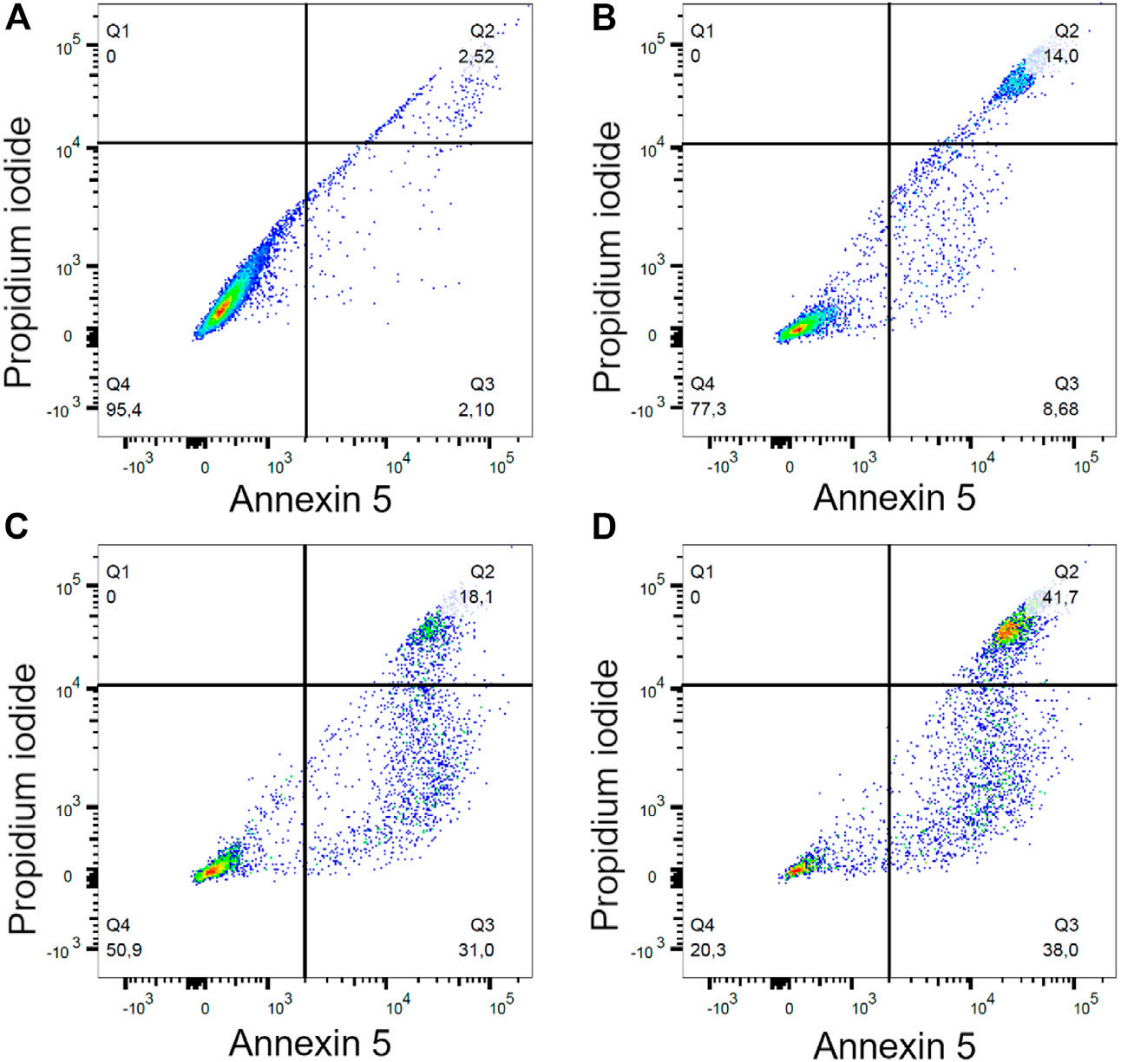
Gating strategy for annexin V/PI staining for invitro experiment. Control cells **(A)**, Cispatin treatment **(B)**, electrospray only **(C)**, electrospray mediated Cisplatin **(D)**.

To quantify cell viability and cell death *via* apoptosis or necrosis 24 h after the electrospray experiment, Annexin/PI staining was performed on LLC cells. Cisplatin at a 1 nM concentration significantly reduced cell viability compared to the controls (*p* = 0.012). Cell viability further decreased from 82.4 ± 4.4% (Cisplatin no electrospray) to 23.8 ± 3.5%, when Cisplatin was electrosprayed (*p* < 0.0001). Interestingly, electrospray itself also decreased cell viability to 46.2 ± 3.5% compared to the controls (*p* < 0.0001) (Figure 3A). It is known that Cisplatin causes cell death *via* apoptosis. Cisplatin treatment alone led to 8.7 ± 4.8% apoptotic cells. However, when Cisplatin was electrosprayed, the percentage of apoptotic cells increased to 36.5 ± 5.3% (*p* < 0.005) (Figure 3B). Moreover, 24 h later, the necrotic cell population also increased with electrosprayed Cisplatin 36.70 ± 0.1% vs. 25.63 ± 2.23% electrospray only, and 7.8 ± 0.5% cisplatin only, respectively (*p* < 0.0005) (Figure 3C). A gating strategy for the cells was used for annexin V/PI staining control cells (Figure 4A), cells treated with Cisplatin (Figure 4B), electrospray only (Figure 4C), and Electrospray mediated Cisplatin delivery (Figure 4D).

### Electrospray Mediated Chemotherapy Significantly Reduces Tumor Volume *in vivo*

**FIGURE 5:**
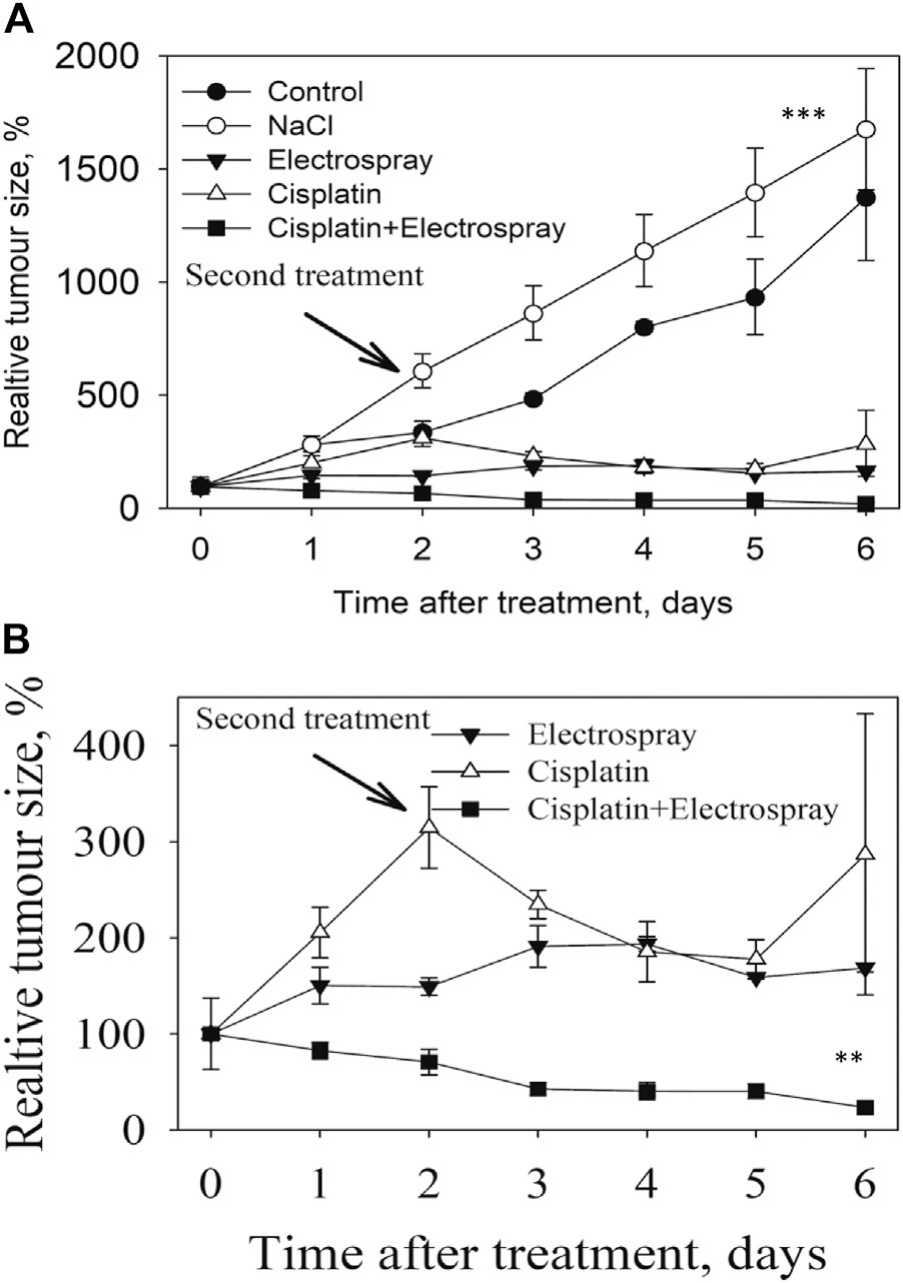
Tumor volume measured in different experimental groups **(A)**. Tumor volume of the three treated groups for better visualization of data **(B)**.

For the *in vivo* experiment, Electrospray was performed at day 0 and day 2, and the subcutaneous tumor growth dynamics were measured daily after *in vivo* electrospray using a caliper. Three days after the first electrospray application, the tumor volume decreased by 42.9% (38 ± 7.3 mm^3^) of the initial volume in response to Cisplatin electrospray, whereas the tumor size in the control group (no treatment) was 200% increased (487 ± 24 mm^3^) compared to the initial tumor volume. There was an increase of 16.2% (247 ± 15 mm^3^) for the Cisplatin only group and a 27% increase (191 ± 22 mm^3^) of initial tumor volume for the electrospray only group. The second treatment was performed on this day and the tumor volume showed a gradual decrease after electrospray mediated Cisplatin treatment compared to the control groups. At day seven, the group receiving the Cisplatin electrospray had the tumor volume reduced by 81.2% of the original volume (22.4 ± 12.1 mm^3^) (Figure 5A) (*p* < 0.0001). The Cisplatin only group had a decrease of 14.4% (168.6 ± 3.9 mm^3^) (*p* < 0.05) and the electrospray only group had a 15.4% (170.6 ± 39.2 mm^3^) reduction of the initial tumor volume (*p* < 0.05).

The tumor volume at day seven in the control group increased by 200% (542 ± 110.0 mm^3^) (control group), and 310% (1,000 ± 51 mm^3^) in the NaCl group. The data of the three treated groups is visualized and presented in Figure 5B.

### Electrospray Mediated Chemotherapy Increases Caspase-3 Positive Cells in Tumor

**FIGURE 6:**
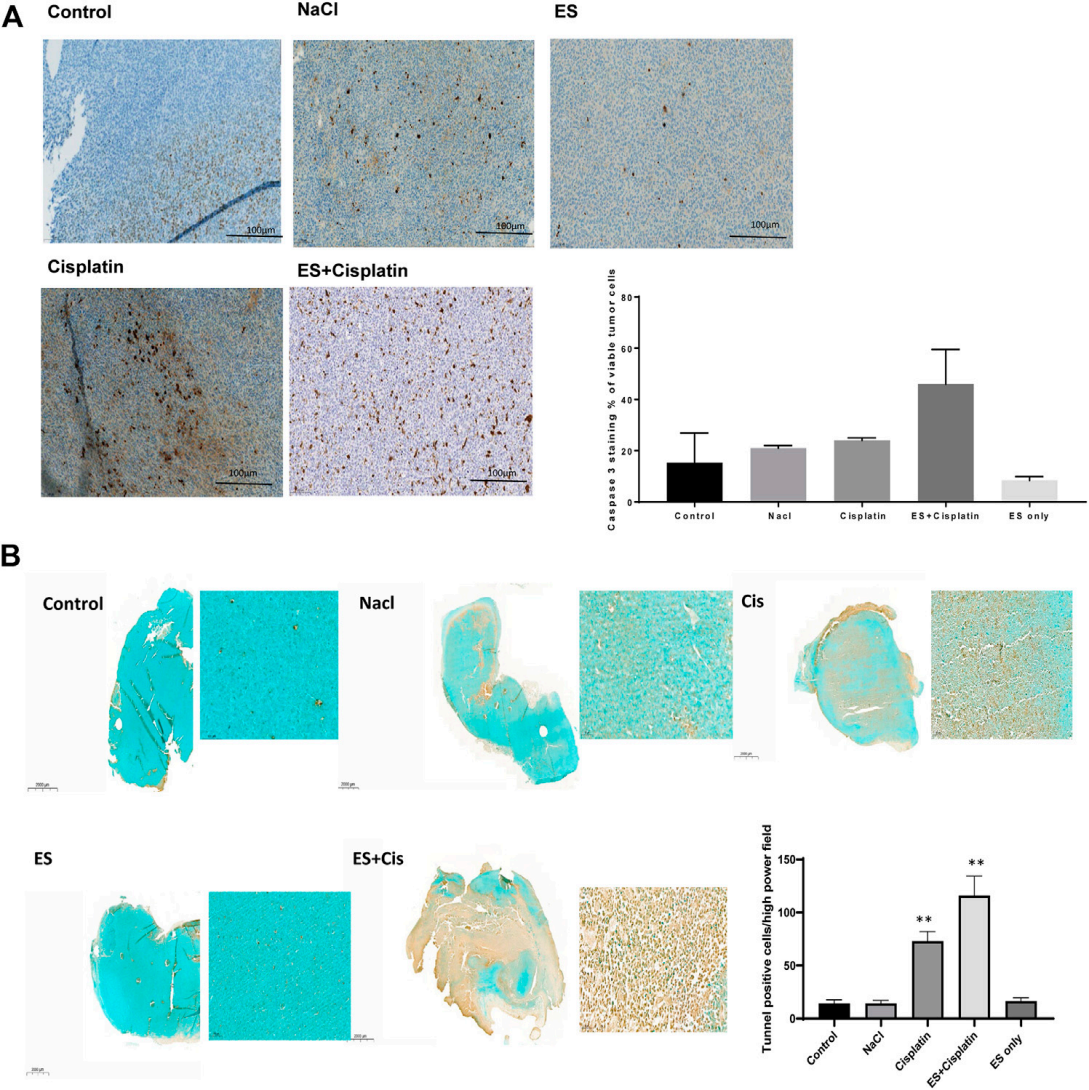
Histological images of caspase 3 staining, and the quantification of caspase 3 positive cells **(A)**. Scale bar 100 µm. Images of tunnel positive cells and quantification of tunnel positive cells **(B)**, scale bar 2,000 µm (entire tumor) and 100 µm for inset images, data is presented as number of cells high power field. Data presented as mean ± SEM, *p* < 0.05*, *p* < 0.001**, *p* < 0.0001 ***.

Increased Capase-3 positive cells were seen in the tumor when Cisplatin was electrosprayed, 44 ± 15%, compared to when Cisplatin was injected into the tumor, 21 ± 1%. Interestingly, fewer positive cells, 15 ± 12%, were observed in the untreated control tumor, when NaCl was injected in the tumor, 21 ± 1%, and when only electrospray was performed, 7.5 ± 1.4% (Figure 6A). In accordance, increased tunnel positive cells were observed; after electrospray mediated Cisplatin 116 ± 18.2%, compared to 73 ± 8.9% after Cisplatin injection. Furthermore, fewer positive cells were observed when NaCl was injected, 14 ± 3%, and 16.4 >% ± 3.14% when only electrospray was performed. The number of positive cells in the absolute control tumors were 14.2 ± 3.5% cells per high power field by photomicroscopy quantification (Figure 6B). Data is presented as the number of cells per high power field.

## Discussion

In the current study, we demonstrate the efficacy of electrospray mediated targeted chemotherapy in a mouse model of lung cancer. Electrospray mediated chemotherapy of Cisplatin is efficient and reproducible and we observed a reduced tumor size after two applications of localized electrospray mediated chemotherapy. Moreover, electrospray mediated Cisplatin induced apoptosis as demonstrated by tunnel positive and Caspase-3 positive cells.

To test for purity and stability of the drug substance during routine drug production, a high performance liquid chromatography (HPLC) is performed for every drug used (Rasmussen et al., 2007). Electrospray is based on the principle of Coulomb repulsion using high voltage (Boehringer et al., 2018), therefore, tandem mass spectrometry was carried out to test the effect of the electrospray process on the stability of Cisplatin. Interestingly, no difference was observed, neither in the ability of Cisplatin to form adducts, nor for changes at the preferred binding site on a DNA oligonucleotide. Following this encouraging result, the effect of electrospray on intra cellular accumulation of Cisplatin and cell death was studied *in vitro*. Intracellular concentration of Cisplatin after electrospray mediated transfer was significantly increased compared to incubation only. Although the exact mechanism of electrospray mediated drug delivery is still under investigation, based on our previous study, we speculate that an alteration in the cell membrane permeability under influence of droplets that are generated and accelerated by an electric field (Boehringer et al., 2018), leads to increased intracellular Cisplatin concentration. This increased intracellular accumulation causes cell apoptosis after electrospray mediated Cisplatin delivery *in vitro*.

Attempts have been made for targeted drug delivery and such studies demonstrate a demand for localized and targeted chemotherapy (Stirland et al., 2013; Senapati et al., 2018). Physical methods like electroporation-based chemotherapy is now applied in clinical practice but are limited to external tumors in the skin, or the head and neck region (Seyed Jafari et al., 2018; Campana et al., 2019). Until now however, no attempt to apply electroporation mediated drug delivery to visceral organs has been reported. Electroporation relies on the application of high voltage, directly on the target organ (Gehl et al., 1999; Graybill and Davalos, 2020), It will therefore generate high electrical current pulses due to the conductivity of the treated tissue, causing power dissipation within the treated area. Moreover, the limiting factor could be the technical difficulties faced in developing a device that can be used to safely apply high voltage inside the body. Therefore, we developed the electrospray-based method where high voltage is applied at the tip of the needle to create spray, and no voltage is applied directly to the target organ (Hradetzky et al., 2012). The electrospray process does not involve the application of high voltage directly at the target organ and is independent of the size of the electrode. The accelerated droplets penetrate the target area probably by making the membrane more permeable. Electrospray is a unique device and is not similar to nebulized inhalers. Nebulizing a chemotherapeutic agent is not targeted, moreover distribution of a nebulized drug is not equal and homogeneous. The electrospray device could, to some extent, be compared to needle free injections (Ravi et al., 2015), with the difference however, that the power source is neither integrated within the device, nor is the drug enclosed inside the electrospray device for a long time as seen with needleless injections.

A significant reduction in the tumor volume was observed after the application of electrospray mediated chemotherapy to the subcutaneous tumor mouse model *in vivo*. As mentioned, the exact mechanism of electrospray is not yet known; nevertheless, a reduction in tumor size was also observed when only electrospray was applied to the tumors, without any chemotherapy agent. This is an additive benefit of the method while targeting tumors. We can only speculate that this effect was due to increased cellular permeability due to electrospray. Moreover, an increase in apoptotic cells after electrospray mediated Cisplatin delivery confirms intracellular entry and also the known mechanism of action of Cisplatin (Siddik, 2003). However, the cell death due to electrospray only without Cisplatin, is significantly lower indicating that electrospray might reduce the tumor by other possible mechanisms that are yet to be explored. Our goal is to develop and modify a device that can be used within the working channel of the bronchoscope as this would enable targeted delivery *via* a minimally invasive method. For this purpose, a miniaturized device design has been elaborated (Fiave et al., 2013) and *in vivo* application is planned. Novel treatment approaches for tumor treatment, like immunotherapy, is one promising approach and it depends on the presence of binding receptors on the tumor surface, but is administered systemically. The heterogeneous distribution and orientation of the receptors on the surface of the tumor is, however, a limiting factor for its success. Electrospray based administration of immunotherapy, directly to the tumor, might help enhance its efficiency and probably reduces the cost of treatment. Similarly, gene therapy approaches for tumor treatment are hindered due to safe and efficient vectors, based on our previous data (Boehringer et al., 2018); we envision the use of electrospray for gene therapy targeting tumors. The electrospray technique is promising and can be used for targeted chemotherapy, gene therapy, and immunotherapy. Moreover, with some design modifications it can be used in different organs, both externally and for internal visceral organs.

The limitations of the current study are that it is a proof-of-concept study and only short-term experiments were performed. A study evaluating the long term effects is warranted to elucidate the effect of electrospray mediated chemotherapy on tumor recurrence. Since it was a feasibility study, we did not perform a control group where the chemotherapeutics were administered systemically due to 3R regulations. However, a study using a large animal model will be a better approach to further validate the data obtained in the current study. In conclusion, electrospray is a promising technique for localized and targeted chemotherapy. With further modifications of the device and application parameters, electrospray could be a very useful tool for minimally invasive localized therapy of intraluminal tumors, using a bronchoscope.

## Data Availability

The raw data supporting the conclusions of this article will be made available by the authors, without undue reservation, to any qualified researcher.
